# Correlation between trait emotional intelligence and prefrontal activation during a verbal fluency task: A functional near-infrared spectroscopy study

**DOI:** 10.1097/MD.0000000000034418

**Published:** 2023-07-21

**Authors:** Takamasa Fukumoto, Haruka Amitani, Ryusei Nishi, Midori Wada, Naoya Oishi, Akihiro Asakawa

**Affiliations:** a Department of Psychosomatic Internal Medicine, Kagoshima University Graduate School of Medical and Dental Sciences, Kagoshima, Japan; b Medical Innovation Center, Kyoto University Graduate School of Medicine, Sakyo-ku, Kyoto, Japan.

**Keywords:** anxiety, depressive symptoms, functional near-infrared spectroscopy, trait emotional intelligence, verbal fluency task

## Abstract

Stress is inevitable in humans and stress changes our physical and mental states. Stress has been studied epidemiologically, biologically, and psychologically. First defined in 1990, emotional intelligence (EI) affects psychological stress management. In contrast, the prefrontal cortex (PFC) is suggested to play a vital role in stress management. Human PFC activity can be inferred from the balance of oxygenated and deoxygenated hemoglobin in cerebral blood flow, which can be measured and calculated using functional near-infrared spectroscopy (fNIRS). An important cognitive activation task to activate the PFC is the verbal fluency task (VFT). Therefore, if the PFC is activated by the VFT and monitored by fNIRS, and the activity correlates with EI, fNIRS can be used to measure EI. In this study, Psychological tests using the self-rating depression scale, state-trait anxiety inventory (STAI), and trait emotional intelligence questionnaire-short form (TEIQue-SF) were conducted to evaluate the correlation with VFT performance. Relative oxygenated and deoxygenated hemoglobin concentrations were measured using an fNIRS device, and their correlation with VFT performance was tested. Spearman correlation coefficient was used to determine correlations. Results were as follows. Although VFT performance did not correlate with the oxygenated hemoglobin concentration ([Oxy-Hb]) changes, [Oxy-Hb] was elevated in all channels. VFT performance was not significantly correlated with the Zung self-rating depression scale (ρ = 0.063, P = .759), trait anxiety or anxiety level as a personal characteristic of STAI (ρ = 0.243, P = .232), state anxiety or anxiety about an event of STAI (ρ = −0.138, P = .500), and TEIQue-SF (ρ = 0.303, P = .132). Healthy individuals PFC activity is not severely affected by their mental state and cognitive activation successfully activates the PFC, not supporting the hypothesis that EI is correlated with frontal cortical activation during the VFT in a nonclinical population. EI may play a vital role in reducing stress associated with depression and anxiety in our social lives. Although we failed to show a statistical correlation between TEIQue-SF and [Oxy-Hb] due to a sample size shortage, our preliminary study was the first to attempt to show the PFC activity of EI through a hemodynamic response. Future research may elucidate the role of EI in reducing psychological stress in social life.

## 1. Introduction

Stress is one of the main factors that change our physical and mental states. Because stress can lead to homeostatic and metabolic regulation dysfunction, it is inevitable for human survival. Traditionally, stress has been researched using 3 approaches: epidemiological, psychological, and biological.^[1]^ However, these approaches cannot be delineated. Psychological stress can trigger negative emotions, which perturb biological homeostasis, such as the hypothalamic–pituitary–adrenal axis.^[1]^ Moreover, psychological stress is often associated with the social environment. During the coronavirus disease pandemic, it was suggested that stress, anxiety, and depression levels were significantly higher in individuals aged 21 to 40 years.^[2]^

Emotional intelligence (EI) affects stress management. In 1990, Salovey and Mayer defined EI as “the ability to monitor one’s own and others’ feelings and emotions, to discriminate among them and use this information to guide one’s thinking and actions”.^[3]^ EI can be divided into 3 categories; ability EI, trait EI, and mixed EI. While ability EI measures the ability to understand emotions and their functions through objective tests rather than participant self-reports, trait EI is assessed by the participants subjective self-reported items,^[4]^ because emotionally intelligent individuals can understand what brought about some of their emotions (e.g., happiness, angriness, sadness) and are able to manage such emotions. Previous research has shown that high trait EI is associated with individual job satisfaction and productivity.^[4]^ There are several measures to assess EI, and Bru-Luna et al^[5]^ recently conducted a systematic review of EI measures and concluded that the trait emotional intelligence questionnaire (TEIQue) is the leading measure of trait EI. The TEIQue has a variety of translated versions with good internal consistency. As mentioned earlier, psychological stress and its management can be partly predicted by EI, using measures such as the TEIQue.

On the contrary, the brain’s prefrontal cortex (PFC) is a region of higher-order functionality, and ventromedial PFC controls emotional responses. When an individual experiences psychological stress, PFC regulation is affected by activating stress pathways through catecholamine release.^[6]^ Recently, anxiety induced by chronic stress has been shown to dysregulate the medial PFC’s control over the basolateral amygdala in rodents, suggesting that the PFC plays a vital role in stress management.^[7]^

In humans, PFC activity can be inferred from the cerebral blood flow (hemodynamics). Cerebral hemodynamic activity was measured and calculated using functional near-infrared spectroscopy (fNIRS). Briefly, the calculated balance between oxygenated and deoxygenated hemoglobin on fNIRS reflects the hemodynamics of the region of interest, such as the PFC. fNIRS has been widely applied in emotion recognition because of its noninvasiveness^[8]^; therefore, it can be inferred that EI can correlate with the PFC’s hemodynamic activity. Moreover, the forehead region, including the PFC, is accessible using fNIRS. Our previous review suggested that cognitive activation during the verbal fluency task (VFT) can be used to assess PFC activity.^[8]^

VFT assesses cognitive ability. Periáñez et al^[9]^ recently compared the VFT and other tests, including the Stroop test, and suggested that the VFT only significantly correlated with the Stroop color–word (SCWT; Pearson coefficients of r = −0.21) and digit symbol substitution tests in the Wechsler adult intelligence scale (4^th^ edition; Pearson coefficients of *R* = 0.22). Moreover, Tran et al^[10]^ recently compared the VFT with the SCWT for the detection of schizophrenia by the change in hemoglobin concentration obtained from fNIRS and showed that the area under the receiver operating characteristic curve of the VFT was higher than that of the SCWT in all regions of interest (ventrolateral PFC, dorsolateral PFC, frontopolar PFC, and orbitofrontal cortex), suggesting that the VFT has higher sensitivity and specificity than the SCWT.

In summary, if EI assessed by the TEIQue reflects the ability to manage psychological stress, and psychological stress intensity can be inferred by measuring the PFC’s hemodynamic response using fNIRS, then EI can be monitored using fNIRS, which enables noninvasive real-time monitoring. To the best of our knowledge, there have been no reports on the real-time monitoring of EI. To achieve noninvasive monitoring of psychological stress, anxiety, and depression, we studied the correlation between psychological and neurophysiological measurements.

## 2. Methods

### 2.1. Participants

This study was conducted at the Department of Psychosomatic Internal Medicine, Kagoshima University Graduate School of Medical and Dental Sciences, Kagoshima, Japan after approval from the Institutional Review Board of Kagoshima University Hospital (approval number:170172). Written informed consent was obtained from all participants before their participation in the study. The inclusion criteria were as follows: Male sex; Age between 20 and 39 years, and; Absence of psychiatric symptoms. Participants with psychiatric and physical symptoms were excluded from the study after evaluation by experienced psychosomatic internal medicine doctors. Therefore, at the time of enrollment, none of the participants had a medical history of major psychiatric disorders, including major depressive disorder, anxiety disorder, neurological disorder, drug dependence, major head injury, major physical illness, or a history of psychotropic medication use. Recruitment was performed using posters, and all candidates who met the inclusion criteria were enrolled. Because this was a preliminary study, we recruited 30 healthy men (mean age 28 ± 4.71 years) without sample size estimation.

### 2.2. Evaluation of depressive symptoms, anxiety, and trait emotional intelligence

Subjective depressive symptoms were evaluated using the Zung self-rating depression scale (SDS), a self-reported questionnaire comprising 20 items. The SDS was developed by Zung in 1965.^[11]^ The scores ranged from 20 to 80, with higher scores indicating a greater degree of depressed mood; the score is a quantitative measure of depression.

Anxiety levels were assessed using the state-trait anxiety inventory Form X.^[12]^ The STAI-X is a self-administered questionnaire that measures the following types of anxiety: State anxiety or anxiety about an event (STAI-S) and; Trait anxiety or anxiety level as a personal characteristic (STAI-T). It consists of 40 questions (20 each for state and trait anxiety). The scale is rated on a 4-point Likert scale. Higher scores indicate greater anxiety.^[12]^

To assess individual subjective EI, we measured trait EI using the Trait Emotional Intelligence Questionnaire-Short Form (TEIQue-SF). We used the validated Japanese version of the TEIQue-SF.^[13]^ The TEIQue-SF comprises 30 items selected from 153 items in the original TEIQue. The global trait EI score was first calculated by averaging the score for each item on a 7-point Likert scale ranging from 1 (completely disagree) to 7 (completely agree). Subscale scores of the 4 factors (Well-Being, Self-Control, Emotionality, and Sociability) were derived from 6, 6, 8, and 6 of the 30 items, respectively.^[14]^

### 2.3. Cognitive activation

**Figure 1.**
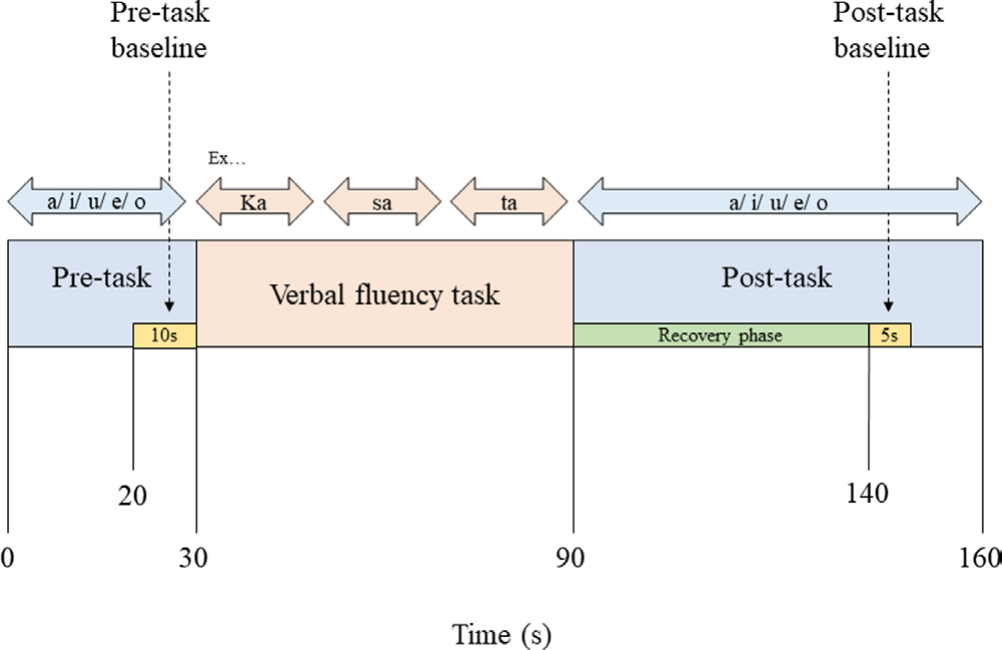
Verbal fluency task protocol.

As mentioned previously, we measured the hemodynamic response of the PFC during cognitive activation using the VFT. Our protocol was based on that of Takizawa et al^[15]^ The protocol was specially designed for neuropsychiatric assessment, including the fNIRS.^[16]^ Figure 1 shows the timeline of the cognitive activation task. During the task, each participant was seated on a comfortable chair in a quiet room without speaking, and was instructed to fix the head position as best as possible. Following 30 seconds of a pre-task period, 60 seconds of the VFT was performed before a 70-second post-task period. The first 50 seconds of the post-task period was defined as the recovery phase. The participants initially repeated the Japanese vowels/a/, i/, u/, e/, and o/, followed by the generation of as many words as they could using the initial designated syllables/ka/, sa/, etc. The 3 initial syllables were changed every 20 seconds during the 60 seconds of the VFT. The total number of correct words was recorded as the task performance for each individual. During the pre- and post-task periods, participants were instructed to repeat the vowels/a/, i/, u/, e/, and o/ at the conclusion of the task (Fig. 1).

### 2.4. fNIRS evaluation of verbal fluency

**Figure 2.**
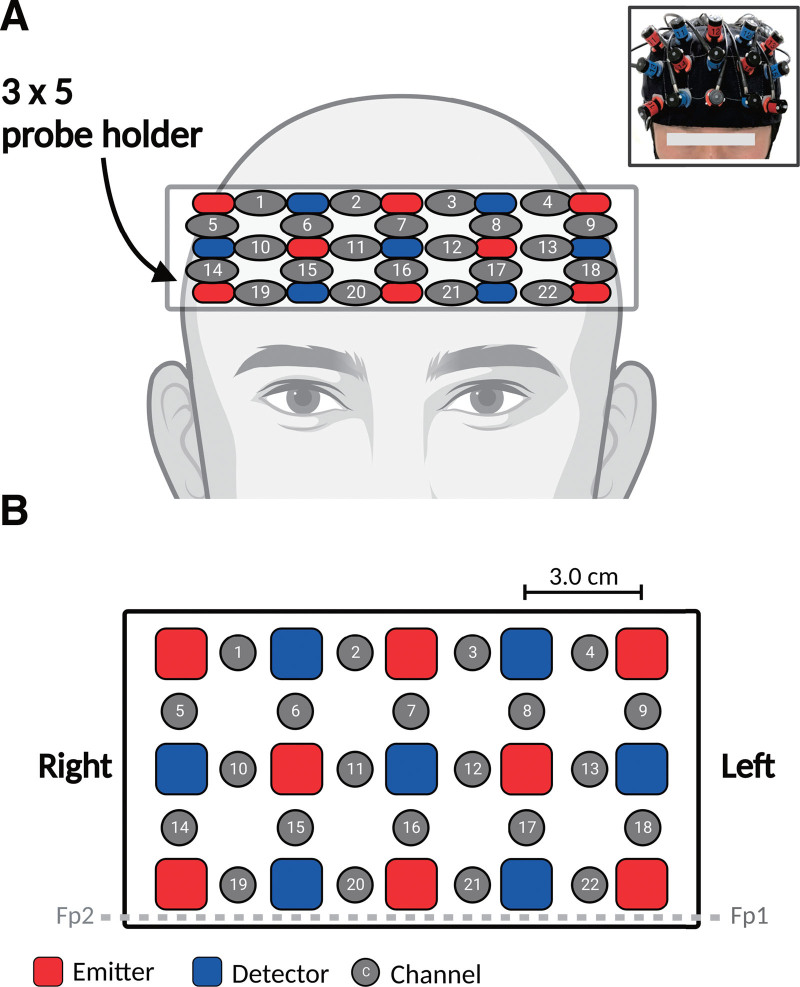
Probe settings and measurement points used by the 22-channel fNIRS device. The probes are placed over the participant’s frontal region bilaterally (A). Precise locations of the probes (B). fNIRS = functional near-infrared spectroscopy.

We used the fNIRS system ETG-4100 (FUJIFILM Corporation, Japan) with near-infrared light at 2 wavelengths (695 nm and 830 nm). Figure 2 shows the probe placement. The 3 × 5 probe holder had 15 electrodes comprising 8 emitters and 7 detectors placed every 3 cm, equivalent to the skull to the surface of the cerebral cortex.^[17,18]^ A channel was defined as the area between 2 electrodes. Therefore, the probe holder had 22 channels, indicating that the hemodynamic responses of 22 different brain regions of interest could be monitored noninvasively. In the brain, the points Fp1 and Fp2 were determined according to the international 10/20 system used in electroencephalography, and the probe holder was positioned such that its bottom line matched the Fp1 to Fp2 line.

The relative changes in oxygenated and deoxygenated hemoglobin concentrations were calculated 10 times per second. The entire measurement time was 160 seconds, which is equivalent to 1600 samples. The pre-task baseline was determined as the mean over a 10-second period just prior to the task period, and the post-task baseline was determined as the mean over the last 5 seconds of the post-task period.^[19]^ Because the baseline fluctuates, we were required to calculate the theoretical baseline per sample. To calculate the theoretical baseline, the fNIRS system builds a linear regression model from the pre- and post-task samples to minimize the root mean squared error. The concentration of oxygenated hemoglobin ([Oxy-Hb]) was measured considering the theoretical baseline as the origin. We averaged 600 samples of [Oxy-Hb] per participant per channel during VFT stimulation. The average obtained was considered as the [Oxy-Hb] elevation induced by VFT stimulation. If the obtained [Oxy-Hb] data were produced from motion artifacts, those data were excluded from the analysis. The entire process was performed automatically using an ETG-4100 instrument (FUJIFILM Corporation, Japan).

### 2.5. Data analyses

Spearman correlation coefficient was used to determine the correlation between [Oxy-Hb] changes in the psychological tests (SDS, STAI-X, and TEIQue-SF) and VFT performance. The significance level was set at *P* < .05. If the calculated *P* value was below the threshold, the calculated Spearman coefficient ρ were considered statistically significant. All statistical analyses were performed using the IBM SPSS Statistics version 25 (IBM Corp. Released 2017. IBM SPSS Statistics for Windows, Version 25.0. Armonk, NY: IBM Corp.).

## 3. Results

### 3.1. Psychological tests in healthy individuals

Owing to an experimental setting error, 4 participants data were excluded from the analysis. Therefore, a total of 26 participants (aged 28.4 ± 4.9 years, mean ± standard deviation) data were analyzed. Table 1 shows individual psychological test scores. The mean and standard deviation of each psychological test and VFT performance were as follows: SDS, 34.4 ± 6.3; STAI-S, 33.7 ± 7.9; STAI-T, 40.5 ± 8.5; TEIQue-SF, 4.6 ± 0.6; and VFT performance, 16.6 ± 4.2.

**Table 1:** Individual psychological tests and VFT performance.

Age	Psychological tests				VFT performance
	SDS	STAI-S	STAI-T	TEIQue-SF	
31	42	37	45	4.5	9
35	34	45	39	5.1	14
31	49	59	61	3.9	20
31	45	42	50	4.3	16
31	32	35	44	4.9	15
37	33	27	41	5.4	21
26	44	25	46	3.7	22
25	25	21	22	6.1	18
25	38	38	43	4.5	15
24	34	31	55	4.3	24
23	33	36	46	4.2	18
24	31	27	30	5.0	24
25	35	39	44	3.9	20
35	37	32	41	3.9	8
31	35	36	39	4.7	13
33	33	31	40	4.5	18
30	28	29	28	5.2	16
35	29	24	36	4.3	13
23	27	31	39	4.6	11
26	39	29	44	3.1	15
25	30	41	33	5.1	15
25	37	29	38	4.4	14
38	36	38	37	5.5	17
23	27	32	38	4.7	14
24	23	26	27	5.1	18
22	39	37	48	4.9	23

SDS = Zung Self-rating Depression Scale, STAI-S = State-Trait Anxiety Inventory Scale Trait anxiety, STAI-T = State-Trait Anxiety Inventory Scale State anxiety, TEIQue-SF = Trait Emotional Intelligence Questionnaire-Short Form, VFT = verbal fluency task.

### 3.2. Correlation between psychological tests and task performance

VFT performance was not significantly correlated with the SDS (ρ = 0.063, *P* = .759), STAI-T (ρ = 0.243, *P* = .232), STAI-S (ρ = −0.138, *P* = .500), or TEIQue-SF (ρ = 0.303, *P* = .132) scores.

### 3.3. Changes in [oxy-Hb] changes during cognitive activation

**Figure 3.**
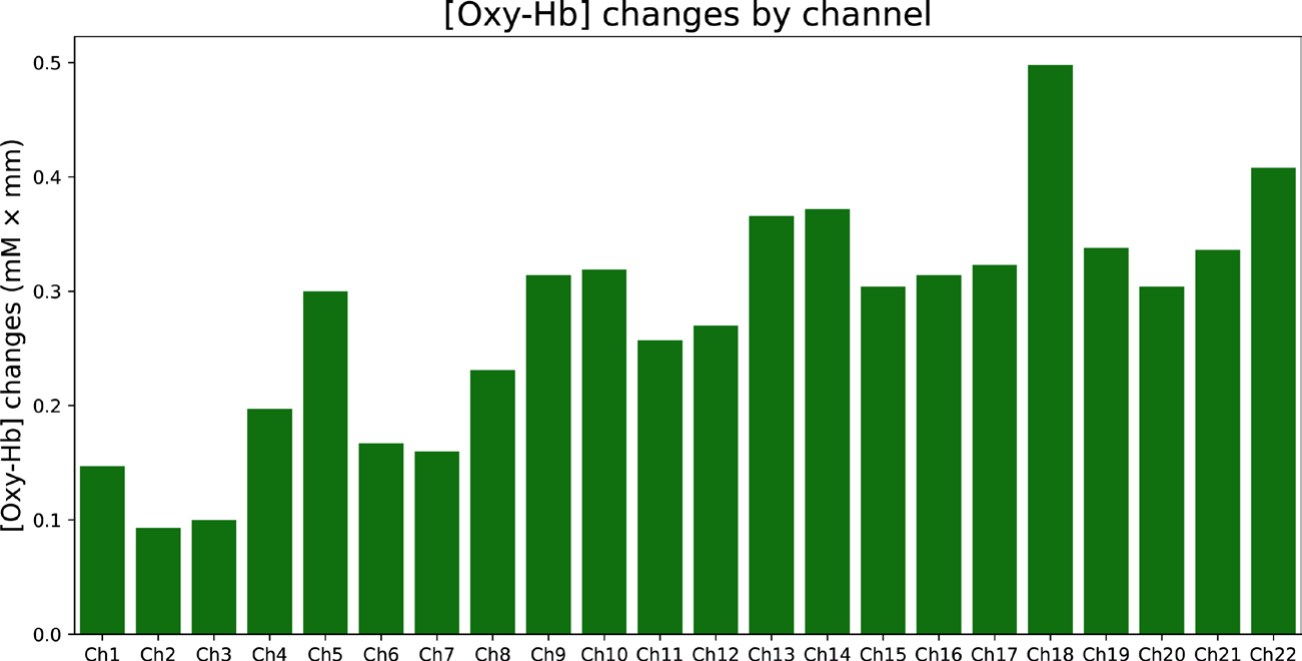
Change in oxygenated hemoglobin concentration by cognitive activation. Each oxygenated hemoglobin concentration change per channel were as follows; #1: 0.147, #2: 0.093, #3: 0.100, #4: 0.197, #5: 0.300, #6: 0.167, #7: 0.160, #8: 0.231, #9: 0.314, #10: 0.319, #11: 0.257, #12: 0.270, #13: 0.366, #14: 0.372, #15: 0.304, #16: 0.314, #17: 0.323, #18: 0.498, #19: 0.338, #20: 0.304, #21: 0.336, #22: 0.408 (each channel number is presented after #).

Figure 3 shows that [Oxy-Hb] changes according to the channel. Although [Oxy-Hb] changed, the change was not significantly correlated with VFT performance (Table 2).

**Table 2:** Correlation between psychological tests and oxygenated hemoglobin changes.

Channels	SDS		STAI-T		STAI-S		TEIQue-SF		VFT performance	
	ρ	*P* value	ρ	*P* value	ρ	*P* value	ρ	*P* value	ρ	*P* value
1	−0.367	.065	−0.258	.203	−0.512	.008	0.124	.547	0.092	.656
2	−0.278	.169	−0.180	.379	−0.401	.042	−0.192	.348	0.013	.950
3	−0.391	.048	−0.321	.109	−0.449	.021	0.016	.939	0.166	.418
4	−0.471	.015	−0.406	.040	−0.311	.122	0.016	.936	0.074	.721
5	−0.464	.017	−0.338	.091	−0.466	.017	0.093	.651	−0.090	.661
6	−0.354	.076	−0.213	.295	−0.341	.088	−0.030	.844	0.091	.658
7	−0.371	.062	−0.258	.204	−0.414	.035	−0.033	.875	−0.078	.705
8	−0.349	.080	−0.378	.057	−0.323	.108	0.023	.911	0.122	.553
9	−0.457	.019	−0.377	.058	−0.284	.160	0.029	.886	−0.051	.804
10	−0.455	.019	−0.303	.132	−0.364	.068	0.021	.919	0.056	.787
11	−0.385	.052	−0.295	.144	−0.353	.077	−0.003	.989	−0.056	.787
12	−0.354	.076	−0.258	.204	−0.302	.133	0.040	.846	0.079	.701
13	−0.362	.069	−0.254	.211	−0.388	.050	0.029	.886	0.132	.522
14	−0.491	.011	−0.305	.130	−0.376	.058	0.185	.367	0.085	.679
15	−0.461	.018	−0.378	.057	−0.423	.031	−0.038	.855	0.029	.889
16	−0.405	.040	−0.401	.042	−0.420	.033	0.057	.783	−0.012	.955
17	−0.337	.093	−0.298	.139	−0.383	.053	0.035	.864	0.062	.763
18	−0.278	.168	−0.144	.482	−0.167	.415	0.076	.713	0.133	.517
19	−0.431	.028	−0.327	.102	−0.200	.328	0.072	.728	0.120	.559
20	−0.415	.035	−0.295	.144	−0.398	.044	0.104	.614	−0.022	.916
21	−0.336	.093	−0.153	.456	−0.284	.160	0.194	.342	0.302	.134
22	−0.330	.136	−0.115	.574	−0.228	.262	0.201	.324	0.205	.316

Statistically significant values and their *P* values are indicated in bold.

SDS = Zung Self-rating Depression Scale, STAI-S = State-Trait Anxiety Inventory Scale Trait anxiety, STAI-T = State-Trait Anxiety Inventory Scale State anxiety, TEIQue-SF = trait emotional intelligence questionnaire-short form, TEIQue = Trait Emotional Intelligence Questionnaire-Short Form, VFT = verbal fluency task.

### 3.4. Correlation between [Oxy-Hb] changes and psychological tests

**Figure 4.**
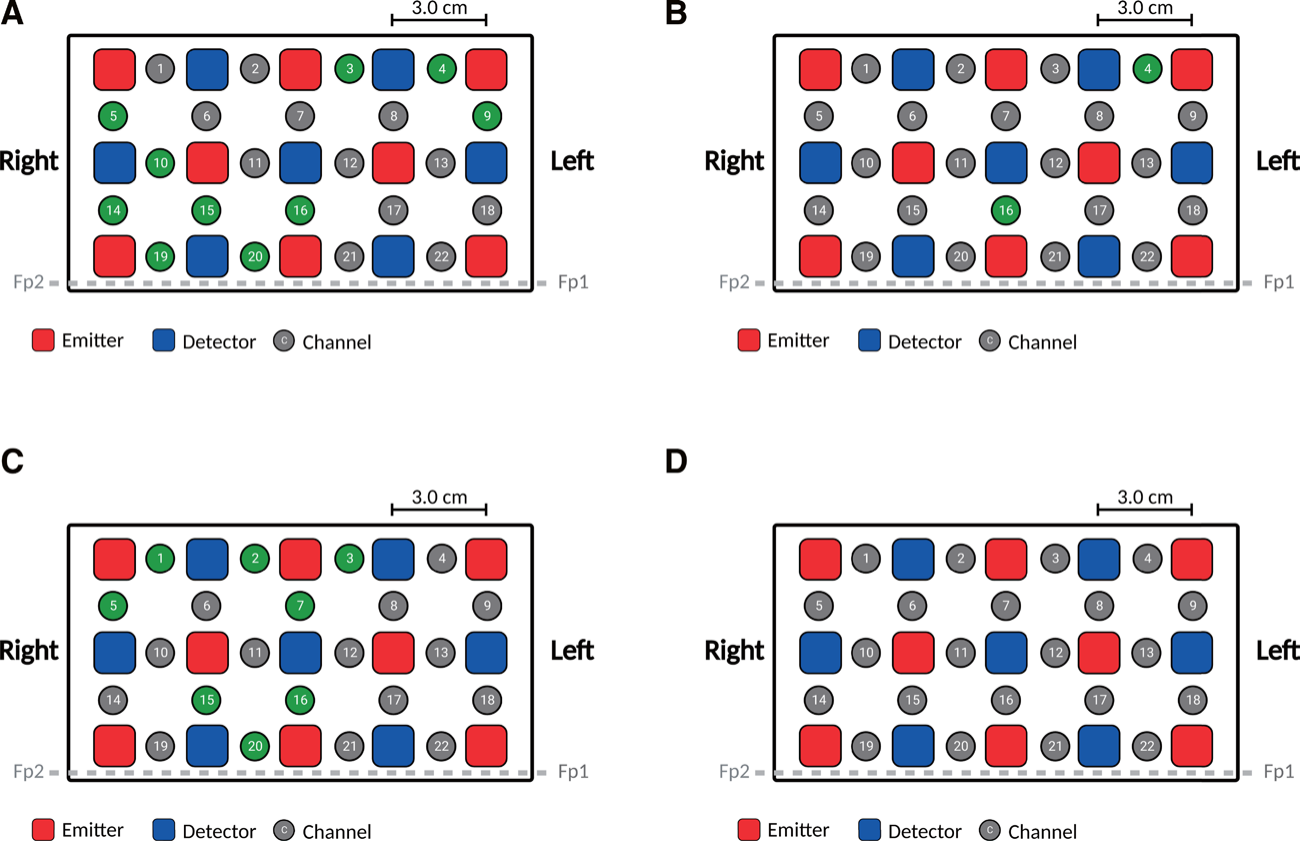
Correlation between psychological tests and changes in oxy-Hb changes. Correlations between psychological tests (SDS, STAI-T, STAI-S, and TEIQue-SF) and [Oxy-Hb] was displayed according to the probe setting. The green channel indicates a significant correlation between the [Oxy-Hb] changes and (A) SDS, (B) STAI-T, (C) STAI-S, and (D) TEIQue-SF. [Oxy-Hb] = oxygenated hemoglobin concentration, SDS = Zung self-rating depression scale, STAI = state-trait anxiety inventory, STAI-S = state anxiety or anxiety about an event of STAI, STAI-T = trait anxiety or anxiety level as a personal characteristic of STAI, TEIQue-SF = trait emotional intelligence questionnaire-short form.

Table 2 shows the correlation between [oxy-Hb] changes and psychological test results. The significantly correlated channels for each psychological test are shown individually (Fig. 4). The SDS scores were negatively correlated with [Oxy-Hb] changes in channels 3, 4, 5, 9, 10, 14, 15, 16, 19, and 20. STAI-T scores were negatively correlated with changes in [Oxy-Hb] in channels 4 and 16. STAI-S scores were negatively correlated with [Oxy-Hb] changes in channels 1, 2, 3, 5, 7, 15, 16, and 20. The TEIQue-SF scores were not significantly correlated with [Oxy-Hb] changes in any channel.

## 4. Discussion

VFT performance did not correlate with anxiety, depression, or EI, suggesting that healthy individuals PFC activity is not severely affected by their mental state. Moreover, higher VFT performance did not indicate higher PFC activity, suggesting that cognitive activation successfully activated the PFC, but the activation could not be quantified by cerebral hemodynamics alone. Our findings are consistent with previous research,^[20]^ which indicated that VFT performance did not have a significant main effect on [Oxy-Hb] changes. On the contrary, [Oxy-Hb] changes in the PFC and VFT performance of healthy individuals were greater compared to those in patients with bipolar disorder,^[21]^ major depressive disorder,^[16]^ obsessive-compulsive disorder,^[22]^ and schizophrenia.^[23]^ Therefore, although greater [oxy-Hb] changes do not imply better VFT performance, psychiatric patients often show lower [oxy-Hb] changes with lower VFT performance than healthy individuals. The change in [Oxy-Hb] was negatively correlated with the SDS, STAI-T, and STAI-S scores, suggesting that anxiety and depression have a negative impact on VFT performance, which is consistent with our previous findings. Moreover, structural changes have recently been suggested to be involved in the development of abnormal depression and anxiety-associated circuits.^[24,25]^

However, the TEIQue-SF showed no statistical correlation with [Oxy-Hb] changes or VFT performance. Therefore, in our study, EI was not associated with PFC activity. Takeuchi et al^[26]^ indicated that the mean diffusivity in the dorsolateral PFC of a magnetic resonance image is significantly correlated with trait emotional intelligence (TEI). TEI involves at least 3 neural circuits of the social cognition network, somatic marker circuitry, and dopaminergic systems, and the social cognition network includes the medial PFC, and the somatic marker circuitry includes the ventromedial PFC.^[26]^

The middle and superior frontal gyri are involved in mentalizing. Mentalization refers to the ability to understand the mental state of oneself and that of others. The left cerebral hemisphere also plays a significant role in mentalizing.^[27]^ Because of the similarity between mentalizing and EI, mentalizing ability can be measured by EI.^[28]^ If the trait EI was significantly correlated with frontal cortical activation during VFT in healthy men, the role of PFC function in mentalizing was indicated. However, we failed to find any statistically significant correlations. The main reason for this may be the small sample size. Further large-scale studies with adjustments for multiple comparisons are required to confirm our hypotheses. To perform a Spearman correlation analysis with a statistical power of 0.8, a significance level of alpha of 0.05, and an effect size of 0.3, we need 167 participants. In this study, we indicated the necessity for further investigation of the correlation between TEI and hemodynamic responses, which will facilitate stress management in healthy individuals. Turner et al^[29]^ suggested the contribution of psychological stress to health via changes in stress reactivity toward the sympathetic–adrenal–medullary system and hypothalamic–pituitary–adrenal axis. If the sample size is sufficient, a large dataset can be obtained, and machine learning approaches may facilitate using [Oxy-Hb] changes and EI as biomarkers of real-time noninvasive stress management, as shown in a previous study of patients with major depressive disorder.^[30]^

Other limitations of our study are as follows: We excluded women from this study to avoid the possible effect of menstruation-induced mood fluctuations on our results as the effect is controversial.^[31–33]^ Therefore, our findings may not apply to women; Psychological stress factors were evaluated based on self-rated scores. We did not evaluate participants psychological stress based on a structured diagnostic interview (e.g., a Structured Clinical Interview for Diagnostic and Statistical Manual diagnoses ^[34]^); We did not consider the participants intelligence quotient, which may have influenced the VFT;^[35]^ Because the study was conducted in participants who were able to apply through recruitment posters, there is a possibility that this study population does not clearly represent healthy Japanese males. However, as all candidates who met the inclusion criteria participated in our study, there were no arbitrary selection biases.

## 5. Conclusion

Our preliminary study is the first to attempt to show the activity of the PFC on EI through a hemodynamic response, although there is a critical sample size shortage. EI may play a vital role in reducing stress associated with depression and anxiety in our social lives.

## Acknowledgements

BioRender was used to render the figures (Figure2 and Figure 4) after obtaining a paid license for publication authorization.

## Author contributions

**Conceptualization:** Haruka Amitani, Akihiro Asakawa.

**Data curation:** Takamasa Fukumoto.

**Formal analysis:** Takamasa Fukumoto, Haruka Amitani, Ryusei Nishi, Midori Wada.

**Funding acquisition:** Akihiro Asakawa.

**Investigation:** Takamasa Fukumoto.

**Methodology:** Naoya Oishi.

**Project administration:** Akihiro Asakawa.

**Resources:** Naoya Oishi.

**Supervision:** Haruka Amitani, Ryusei Nishi, Naoya Oishi.

**Validation:** Ryusei Nishi.

**Visualization:** Midori Wada.

**Writing – original draft:** Takamasa Fukumoto.

**Writing – review & editing:** Haruka Amitani, Ryusei Nishi, Midori Wada, Akihiro Asakawa.

## Correction

In the published article, part of the abstract text has now been updated online from “VFT performance was significantly negatively correlated with the Zung self-rating depression scale (ρ = 0.063, P = .759), trait anxiety or anxiety level as a personal characteristic of STAI (ρ = 0.243, P = .232), and state anxiety or anxiety about an event of STAI (ρ = −0.138, P = .500), whereas no correlation was found with the TEIQue-SF (ρ = 0.303, P = .132). Healthy individuals PFC activity is not severely affected by their mental state and cognitive activation successfully activates the PFC, supporting the hypothesis that EI is correlated with frontal cortical activation during the VFT in a nonclinical population” to “VFT performance was not significantly correlated with the Zung self-rating depression scale (ρ = 0.063, P = .759), trait anxiety or anxiety level as a personal characteristic of STAI (ρ = 0.243, P = .232), state anxiety or anxiety about an event of STAI (ρ = −0.138, P = .500), and TEIQue-SF (ρ = 0.303, P = .132). Healthy individuals PFC activity is not severely affected by their mental state and cognitive activation successfully activates the PFC, not supporting the hypothesis that EI is correlated with frontal cortical activation during the VFT in a nonclinical population.”
