# Correlation between trait emotional intelligence and prefrontal activation during a verbal fluency task: A functional near-infrared spectroscopy study: Erratum

**DOI:** 10.1097/MD.0000000000049800

**Published:** 2026-07-17

**Authors:** Takamasa Fukumoto, Haruka Amitani, Ryusei Nishi, Midori Wada, Naoya Oishi, Akihiro Asakawa

In the article “Correlation between trait emotional intelligence and prefrontal activation during a verbal fluency task: A functional near-infrared spectroscopy study,”^[1]^ which appears in Volume 102, Issue 29 of *Medicine*, was originally published with an incorrect data in the abstract. To avoid ambiguity, a part of the abstract text has been revised **from** “VFT performance was significantly negatively correlated with the Zung self-rating depression scale (ρ = 0.063, *P* =.759), trait anxiety or anxiety level as a personal characteristic of STAI (ρ = 0.243, *P* =.232), and state anxiety or anxiety about an event of STAI (ρ = −0.138, *P* =.500), whereas no correlation was found with the TEIQue-SF (ρ = 0.303, *P* =.132). Healthy individuals PFC activity is not severely affected by their mental state and cognitive activation successfully activates the PFC, supporting the hypothesis that EI is correlated with frontal cortical activation during the VFT in a nonclinical population” **to** “VFT performance was not significantly correlated with the Zung self-rating depression scale (ρ = 0.063, *P* =.759), trait anxiety or anxiety level as a personal characteristic of STAI (ρ = 0.243, *P* =.232), state anxiety or anxiety about an event of STAI (ρ = −0.138, *P* =.500), and TEIQue-SF (ρ = 0.303, *P* =.132). Healthy individuals PFC activity is not severely affected by their mental state and cognitive activation successfully activates the PFC, not supporting the hypothesis that EI is correlated with frontal cortical activation during the VFT in a nonclinical population” in the updated version.
